# Intrabone transplant provides full stemness of cord blood stem cells with fast hematopoietic recovery and low GVHD rate: results from a prospective study

**DOI:** 10.1038/s41409-018-0335-x

**Published:** 2018-09-19

**Authors:** Francesca Bonifazi, Elisa Dan, Myriam Labopin, Mariarosaria Sessa, Viviana Guadagnuolo, Martina Ferioli, Simonetta Rizzi, Sabrina De Carolis, Barbara Sinigaglia, Maria Rosa Motta, Andrea Bontadini, Valeria Giudice, Giovanni Martinelli, Mario Arpinati, Michele Cavo, Massimiliano Bonafé, Gianluca Storci

**Affiliations:** 1 Institute of Hematology “L. and A. Seràgnoli”, University Hospital S. Orsola-Malpighi, Bologna, Italy; 2 Hôpital Saint-Antoine 184 rue du Faubourg Saint-Antoine, 75571 Paris Cedex 12, Paris, France; 3 DIMES, Department of Experimental, Diagnostic and Specialty Medicine, University of Bologna, Bologna, Italy; 4 Immunogenetics, University Hospital S. Orsola-Malpighi, Bologna, Italy; 5 Apheresis Unit, University Hospital S. Orsola-Malpighi, Bologna, Italy; 6 Biosciences Laboratory, Istituto Scientifico Romagnolo per lo Studio e la Cura dei Tumori (IRST) IRCCS, Meldola, Italy; 7 Interdepartmental Center “Luigi Galvani”, CIG, University of Bologna, Bologna, Italy

**Keywords:** Medical research, Diseases

## Abstract

Umbilical Cord Blood (UCB) represents a valid option for patients with hematopoietic malignancies lacking an HLA matched donor. To overcome the limitation of the low stem cell dose of UCB, the intrabone (IB) route has been proposed. We report the results of a prospective study on a poor-prognosis cohort of 23 patients receiving intrabone single UCB transplant (Clinicaltrials.gov NCT00886522). Cumulative incidence of hematological recovery at day 90 was 82 ± 9% (ANC > 0.5 × 10^9^/L) and 70 ± 10% (platelet > 50 × 10^9^/L) and correlated with CD34 + cells in the graft. NRM was 20 ±  9%. No severe aGVHD and only one extensive cGVHD occurred, with fast immune reconstitution. To test the hypothesis that the direct IB injection could affect the expression of stem cells regulatory pathways, CD34 + cells from BM aspirates at day + 10, + 20, + 30, processed in hypoxic conditions mimicking the BM-microenvironment (7%pO_2_), were studied for the expression of c-Mpl, Notch1 and CXCR4. We found that the expression of c-Mpl in CD34 + cells at day + 10 significantly correlated with hematological recovery. In conclusion, IB-UCB transplant success is associated with low incidence of GVHD and high-speed platelet recovery; intrabone route may preserve full hematopoietic stemness by direct delivery of UCB stem cells into the hypoxic HSC niche.

## Introduction

Umbilical Cord Blood (UCB) represents a valid option for patients needing an allotransplant but lacking a HLA matched donor. Despite the clear evidence of its efficient anti-leukemic effect [1], delayed engraftment with relatively high non-relapse mortality (NRM) remains the major obstacle to UCB transplant in the adult. Several strategies have been proposed to overcome this limitation, which is likely due to the low hematopoietic stem cell (HSC) dose. Double UCB transplant, one of the most popular option currently used to ameliorate the speed of engraftment, failed to demonstrate formal proof of superiority [2]. Further approaches emerged, such as ex-vivo UCB expansion by means of the administration of cytokines [3], Notch ligands [4], nicotinamide [5] or mesenchymal cells co-culture [6]. Factors other than the cell dose, such as HLA mismatches [7] and the presence of pre-transplant donor-specific anti-HLA antibodies [8], affect UCB engraftment. An alternative approach is to increase the seeding efficiency by bringing more cells to the physiologic place, i.e. the hematopoietic *niche*, as it occurs in direct intrabone (IB) injection of UCB [9]. IB transplant has been safely applied to adult patients with impressive effects on platelet recovery, which is a surrogate marker of transplant outcome. Speculatively, the injection of UCB HSC into the bone marrow (BM) exposes the cells directly to beneficial stimuli provided by the HSC-niche, such as components of the c-Mpl/TPO, Notch/Jagged1, CXCR4/SDF1 receptor/ligand systems [10–12], as well as low pO_2_ level [13]. In principle, all the above HSC niche cues potentially empower UCB-HSC capability to recover hematopoiesis [14, 15]. Here we report on a prospective clinical trial (NCT00886522) on patients with very advanced hematological malignancies who underwent allogeneic transplant with one UCB unit given via IB route. Moreover, we show evidence that c-mpl expression in post-transplant CD34 + cells associates with hematopoietic recovery after IB-UCB. Moreover, because we here found that the level of c-Mpl in CD34 + is higher under hypoxic conditions, we surmise that the direct injection of CD34 + in the hypoxic HSC niche may contribute to the success of the IB- procedure.

## Methods

### Study design

This is a single-center, non-randomized, open-label, phase 2 clinical study (Clinicaltrials.gov NCT00886522). The primary endpoint of the study was the rate of hematologic engraftment. The sample size calculation (Fleming’s single step procedure) was made setting the minimum proportion of patients with engraftment at day + 42 for not abandoning the study (p_0_) = 60% and proportion of patients needed to test the main hypothesis in a phase III study (p_1_) = 90%. A minimum of 17 patients were needed. The study was approved by the local Ethics Committee (152/2008/U/Sper). All patients signed a written informed consent. All the authors had access to primary clinical data. Inclusion criteria were: adult patients (18–65 years) suffering from high-risk hematological malignancies, lacking a sibling or an unrelated adult HLA identical donor, receiving a myeloablative conditioning regimen (either busulfan or TBI based conditioning) and graft-vs-host disease (GVHD) prophylaxis with Ciclosporin-A, Micophenolate-Mofetile and 15–30 mg/kg (total dose) of anti-lymphoglobulin (ATLG, Grafalon®, NEOVII Biotech, Gräfelfing, Germany), given from day −6 to −2. Advanced phase or active disease at transplant and previous autologous and/or allogeneic transplants were not exclusion criteria. UCB units were selected according to the HLA minimum standard criteria of the Italian Bone Marrow Donor Registry (http://ibmdr.galliera.it/standard-ibmdr/cosa-sono-gli-standard-operativi-ibmdr) and contained a minimum of 1.5 × 10e^7^/kg nucleated cells.

Hematological recovery, acute and chronic GVHD were graded according to standard criteria.The patients were also evaluated for the immunological recovery after transplant by FACS analysis for CD3, CD4, CD8, CD56, CD19 positive cells and for CD34 and c-mpl expression.

### Intrabone infusion

The selected UCB units were rapidly thawed, washed and re-suspended in albumine-dextran solution as previously described [9]. The recovery of cells after thawing is reported in Supplementary Table I. Infusion was preceded by the administration of analgo-sedation (remifentanil alone or associated with continuous infusion of propofol or midazolan), in the operating room. After the washing procedure, UCB unit was injected directly in the posterior superior iliac crests of the patients lying on a side, after few minutes from the end of the thawing procedure, the content of each syringe was 5–6 ml injected slowly (1–2 min). The procedure, repeated 4–6 times, lasted overall 20–30 min. Thereafter patients were reallocated to their standard single, air-positive pressure rooms with HEPA-filtered air, until discharge.

### In vitro studies

CD34 + cells were immuno-magnetically separated (CD34 Microbeads Kit, Milteny Biotech, Gmbh, Germany) from 11 patients undergoing BM aspirates at 3 time points (day + 10, + 20, + 30) after IB-UCB transplant. CD34 + cells separation was performed under a BM-mimicking pO_2_ concentration (7%pO_2_ [16]) generated in a hypoxia cabinet (In vivo300, Ruskinn^®^, Ruskinn Technology, United Kingdom). The above level of pO_2_ was extremely similar to our measurements of pO_2_ of 11 BM samples obtained by means of blood-gas analysis syringe, i.e., 7.5% pO_2_ + 1.3%pO_2_ (Supplementary Figure 1A). Post-transplant CD34 + cells mRNA expression was evaluated by real time PCR in iCycler iQ™ Real-Time PCR Detection System (Applied Biosystems, Carlsbad, CA, USA). Each sample was analyzed in triplicate. Sets of primers and fluorogenic probes specific for c-Mpl, Notch-1 and CRCX4 mRNAs were purchased from Applied Biosystems. The reactions were incubated at 50 °C for 2 min; 95 °C for 15 min followed by 45 cycles of 95 °C for 15 s and 60 °C for 1 min. The relative amount of the target mRNA was calculated equal to 2− (ΔCt target mRNA− ΔCt control), using human *β*-glucuronidase mRNA as control. All the values were normalized over a sample of normal BM CD34 + cells and expressed as fold increase. The logarithm of ΔΔ*C*t was used to normalize the distribution.

### Statistical analysis

Survival plots were obtained according to Kaplan–Meyer method. NRM, relapse incidence and hematological recovery were calculated by cumulative incidence method with death or second transplant being considered as competing risks. Multivariate analysis of censored data was performed by Cox regression model. Variables analyzed in univariate analysis were: phase, age, ATLG, infused total nucleated cells, CD34 + and CD3 + cells. The variables resulting in a *p*-value > 0.2 were then included in multivariate analyses. All tests were two sided with type I error fixed at 0.05. Statistical analyses were performed with SPSS 19 (SPSS Inc, Chicago, IL, USA) and R 2.13.2 (R Development Core Team, Vienna, Austria) software packages.

## Results

**Fig. 1:**
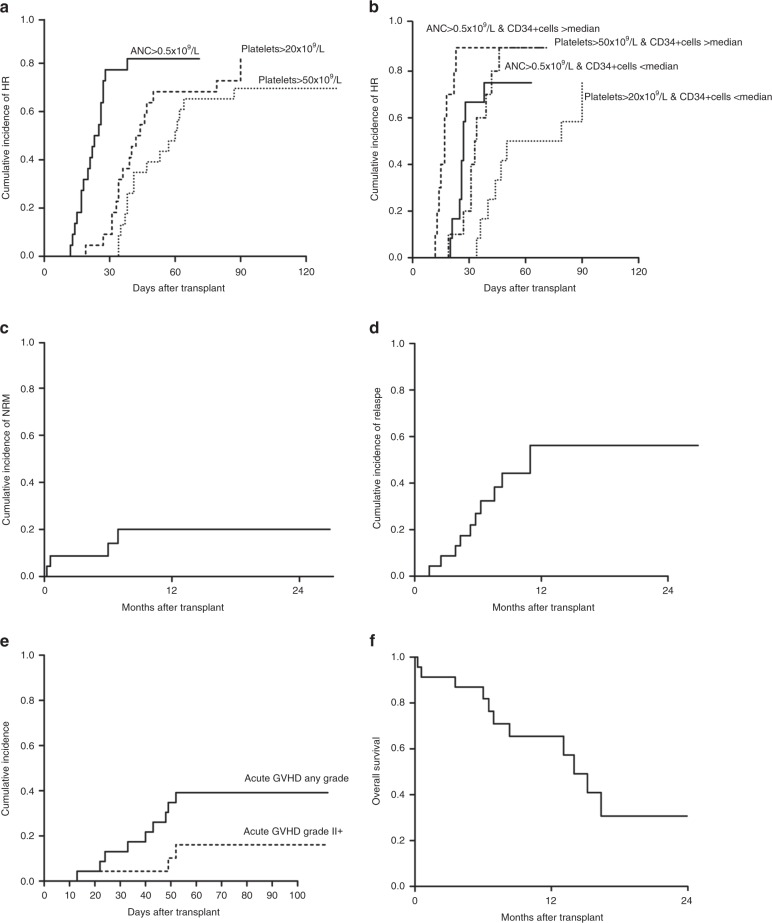
**a** Cumulative incidence of hematologic recovery (HR). Times to neutrophil (ANC) and platelet (Plt) recovery were defined as time from transplant to > 0.5 × 10^9^/L or, 20 × 10^9^/L and 50 × 10^9^/L, respectively. The cumulative incidence of ANC and platelet recovery were calculated counting deaths from other causes as competing risk. **b** Cumulative incidence of HR according to CD34 + cells. The hematologic recovery was significantly associated with CD34 + cells (ANC > 0.5 × 10^9^/L p = 0.04; Platelet > 0.5 × 10^9^/L p = 0.07). Median CD34 + cells infused was 0.54 × 10^5^/kg. **c** Cumulative incidence of non relapse mortality (NRM). The cumulative incidence of NRM was 20  ±9% and was calculated counting deaths from other causes as competing risk. **d** Cumulative incidence of relapse/progression. Cumulative incidence of relapse/progression was 56  ±10%. In the study protocol, therapy after relapse/progression was free. Two relapses were treated with a further IB-UCB transplant: one was a by phenotypic refractory Acute Leukemia, who achieved a stable complete remission lasting 9 months, then he died for leukemia. The second was a Chronic Myeloid Leukemia patient transplanted in blastic phase, relapsing after 6 months from IB-UCB transplant with hematologic relapse, central nervous system involvement and a solid mass in the breast, which completely responded to Ponatinib. She received a second IB-UCB transplant achieving a further complete remission lasting 5 months; however relapse occurred again resulting in death. **e** Cumulative incidence of acute GVHD, **f** Overall survival

Characteristics of patients are reported in Table 1. No severe adverse events related to IB infusion were recorded and the IB-UCB infusion were well tolerated. The median volume injected IB was 27.6 ml (range: 19.0–58.2 ml) via 5 injections in the posterior iliac crests. All patients engrafted but two, who were both re-transplanted with a second IB-UCB unit: one, a non-Hodgkin lymphoma died of aplasia at day 25; the other was a patient with refractory acute myeloid leukemia, engrafted the second IB-UCB transplant, achieving a complete remission that lasted 11 months. Cumulative incidence of hematological recovery (ANC > 0.5 × 10^9^/L, platelet > 20 × 10^9^/L and > 50 × 10^9^/L) at day 90 were 82 ± 9%, 82 ± 9% and 70 ± 10%, respectively (Fig. 1a**)**. Cumulative incidence of neutrophil recovery at day 28 was 77.3 ± 9%. Notably, the amount of CD34 + cells after thawing, which is highly correlated to the number of CD34 + at cryopreservation (data not shown), was significantly associated with hematological recovery, both in univariate and multivariate analysis (Fig. 1b and Supplementary Tables II and III). NRM was 20 ± 9% (Fig. 1c): two patients died of toxicity, one of infection and one of graft failure. CMV reactivation occurred in 13/23 patients with a median onset at day + 33 (range 13–90 days), but no CMV disease was recorded. Relapse incidence was 56 ± 10% (patients in early disease only 21%) (Fig. 1d). aGVHD occurrence is indicated in Fig. 1e: 16 patients had no GVHD, four patients had grade I, two grade II and one grade III (Table I). Only one patient developed extensive chronic GVHD with a median follow up of 45 months. Time to CsA discontinuation was 145 days (range 51–401 days). Any of the clinical factors analyzed (phase, age, ATLG dose, infused total nucleated cells, as wells as CD34 + and CD3 + cells) was found to be associated with GVHD occurrence (Supplementary table II).

**Table 1:** Clinical characteristics of intrabone UCB transplants

No. of patients		23
Years of transplant		2009–2012
Age	Median, range	36 years (16–57)
Gender	Males, %	9 (39%)
Interval diagnosis-transplant	Median, range	10 months (4–60)
Diagnosis		
	AML, *n* (%)	16 (69.6)
	ALL, *n* (%)	4 (17.4)
	CML, *n* (%)	1 (4.3)
	NHL, *n* (%)	1 (4.3)
	MM, *n* (%)	1 (4.3)
Phase at transplant		
	1st Complete Remission (%)	5 (21.7)
	2nd Complete Remission (%)	3 (13.0)
	>2 Complete Remission (%)	3 (13.0)
	Primary Refractory (%)	6 (26.1)
	Resistant Relapse (%)	4 (17.4)
	Progression (for MM) (%)	1 (4.3)
	Blastic Phase (for CML) (%)	1 (4.3)
Previous transplant		
	Autologous, n of pts (%)	6 (26.1)
	Allogeneic, n of pts (%)	3 (13.0)
Courses of therapy	Median, range	4.5 months (2–7)
Conditioning regimen		
	Cy-TBI (%)	3 (13.0)
	Bu- Cy (%)	11 (47.8)
	Bu- Flu-TT (%)	9 (39.2)
GVHD prophylaxis	ATG-CsA-MMF (%)	23 (100.0)
CMV serostatus		
	Recipient Neg/Donor Neg, *n* (%)	3 (13.0)
	Recipient Pos/Donor Neg, *n* (%)	20 (87.0)
Donor recipient HLA matching		
	4/6, *n* (%)	20 (87.0)
	5/6, *n* (%)	3 (13.0)
TNC×10^7^/kg pre thawing	Median, range	3.04 (1.91–5.48)
TNC×10^7^/kg infused	Median, range	2.06 (0.20–3.92)
CD34 + cells×10^5^/kg pre thawing	Median, range	1.29 (0.12–3.46)
CD34 + cells×10^5^/kg infused	Median, range	0.54 (0.23–2.90)
CD3 + cells×10^6^/kg infused	Median, range	25.45 (1.6–5.3)
Vitality	Median, range	91% (37–95%)
Hematologic recovery		
median day to ANC > 0.5 × 10^9^/L	Median day, range	21.5 (12–38)
median day to platelets > 20 × 10^9^/L	Median day, range	39.5 (19–90)
median day to platelets > 50 × 10^9^/L	Median day, range	47 (34–150)
Acute GVHD	Grade I, *n* (%)	4 (17.4)
	Grade II, *n* (%)	2 (8.7)
	Grade III, *n* (%)	1 (4.3)
	Grade IV, *n* (%)	—
Chronic GVHD	Limited, *n* (%)	3 (13.0)
	Extensive, *n* (%)	1 (4.3)

*AML* Acute Myeloid Leukemia, *ALL* Acute Lymphoblastic Leukemia, *CML* Chronic Myeloid Leukemia, *NHL* Non-Hodgkin Lymphoma, *MM* Multiple Myeloma, *Cy* Cyclophosphamide, *TBI* total body irradiation, given unfractioned at low dose-rate, *Bu* i.v. busulfan, total dose 12.8 mg/kg, *Flu* fludarabine, total dose 150 mg/m^2^, *TT* thiotepa, total dose 10 mg/kg, *ATG* antitlymphocyte globulin (Grafalon, NEOVII, Germany), total dose 15–30 mg/kg from day −6 to day −2, *CsA* Ciclosporin-A, given according to early post transplant serum level between 200 and 300 ng/ml, *MMF* micophenolate mofetile, 30 mg/kg bid from + 1 to + 28, *TNC* total nucleated cells, *ANC* absolute neutrophil count, *GVHD* graft vs. host disease, *CMV* cytomegalovirus

**Fig. 2:**
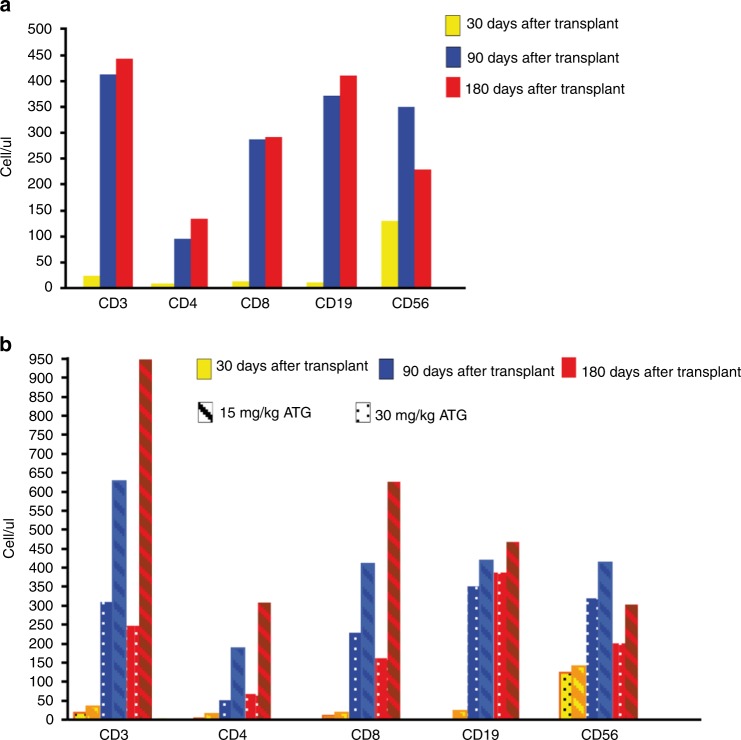
**a** Immune reconstitution after IB-UCB transplant. Recovery of CD3 + , CD4 + , CD8 + , CD19 + and CD56 + cells from peripheral blood samples obtained after 30, 90 and 180 days after IB-UCB transplant. **b** Immune reconstitution after IB-UCB transplant according to ATLG dose. Immune reconstitution from peripheral blood samples according to ATLG dose: patients receiving higher ATLG dose (30 mg/kg) show a more impaired CD3 + , CD4 + and CD8 + cells recovery than that from patients receiving lower dose (15 mg/kg); CD19 + and CD56 + cells recovery didn’t seem to be affected by ATLG dose

**Fig. 3:**
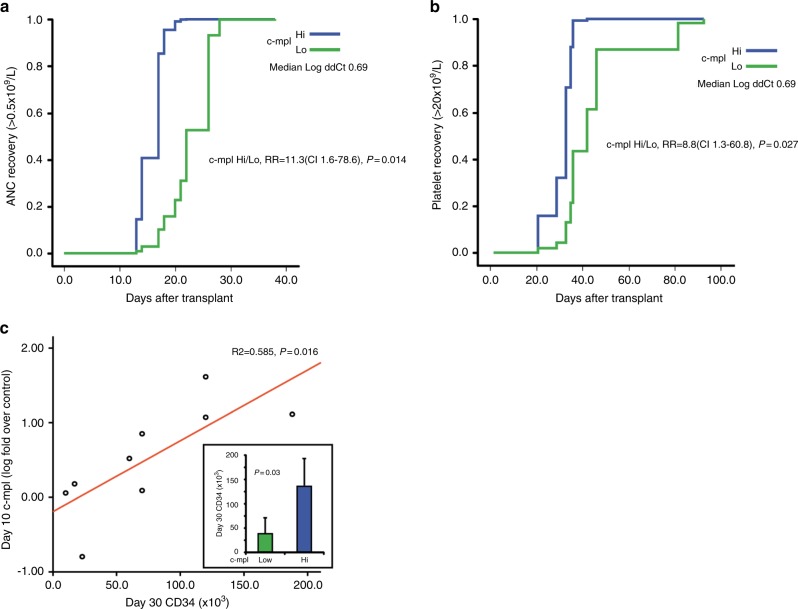
**a** Cumulative incidence of neutrophil recovery according to c-Mpl level evaluated on day + 10 in BM aspirates CD34 + cells processed under hypoxic condition. Time to ANC > 0.5 × 10^9^/L according to + 10 BM-derived CD34 + cells c-Mpl mRNA level (low/high classified respect to the median value of Log ddCT:0.69). **b** Cumulative incidence of platelet recovery according to c-Mpl level evaluated on day + 10 in BM aspirates CD34 + cells. Time to platelet > 20 × 10^9^/L according to + 10 BM-derived CD34 + cells c-Mpl mRNA level (low/high classified respect to the median value of Log ddCT:0.69). **c** Regression analysis of c-Mpl level at + 10 day vs amount of CD34 + cells at + day 30. In the Box window the expression of Low vs Hi of c-Mpl is significantly associated with the capability of in vivo of CD34 + cells measured by the amount of CD34 + cells at day + 30. (Low/hi were classified as below/above the median value of Log ddCT:0.69)

Relapse incidence and overall survival (Fig. 1f) were not significantly associated with any of the analyzed factors (Supplementary Tables II and III). The immune reconstitution following UCB transplant is reported in Fig. 2. Numbers of CD4 + and CD8 + T cells were < 50/ul at 1 month after transplant then increased to > 100/ul within 3 months. Recovery of B cells and NK cells was rapid and full within 3 months after transplant. A dose dependent impairment of CD3 + , CD4 + and CD8 + cell recovery but similar CD56 + and CD19 + counts was observed in patients receiving 30 mg/Kg of ATLG compared to those receiving 15 mg/Kg. To gain an insight into the biology of IB-UCB Transplant, we reasoned that delivering HSC directly into the physiological IB micro-environment could effectively maintain hypoxic signaling thus preserving the engraftment potential [10, 13, 17]. To this purpose we isolated CD34 + under BM-mimicking hypoxic conditions (7%pO_2_ [16] and see supplementary Figure 1a). We observed that the expression level of c-Mpl was significantly higher when CD34 + cells were processed under controlled hypoxia (7% pO_2_) compared to bench-work atmospheric pO_2_ (Supplementary Figure 1B). Accordingly, the percentage of c-Mpl + CD34 + cells in BM samples was higher under hypoxic than atmospheric pO_2_ (Supplementary Figure 1C). We thus reasoned that the processing of BM samples under 7% pO_2_ BM-mimicking pO_2_ may preserve the in vivo expression level of c-Mpl, and thus may allow to evaluate its association with parameters of hematopoietic recovery. Following this reasoning, we performed the separation of CD34 + cells from BM aspirates of eleven patients at three time points (day + 10, + 20, + 30) under 7% pO_2_. Then, CD34 + cells were assessed for the expression of three stem cell regulatory genes, namely: c-Mpl, CXCR4 and Notch1. We found that day + 10, but neither day + 20 nor day + 30, c-Mpl mRNA level was positively correlated with ANC and platelet recovery (Fig. 3a, b). Conversely, day + 10, + 20, + 30 CXCR4 and Notch1 mRNA did not correlate with platelets or ANC recovery (Supplementary Figure 2). The positive correlation between c-Mpl mRNA expression and the hematological recovery was confirmed by multivariate analysis for both ANC recovery (RR = 11.3, CI 95% 1.6–78.6 *p* = 0.014) and platelet recovery > 20 × 10^9^/L (RR = 8.8, CI 95% 1.3–60.8, *p* = 0.027). Moreover, day + 10 c-Mpl mRNA level was correlated with the amount of CD34 + cells at day + 30 (Fig. 3c). With the limitation of the small sample size of the study, these data are supportive that day + 10 c-Mpl expression is significantly associated with both hematologic recovery and the capability of in vivo expansion of HSC after transplant.

## Discussion

The use of UCB transplant has significantly decreased in the last years [18, 19], with important differences across countries, despite its performance has never been demonstrated inferior to other transplant types which are now fastly growing. In the present study, we confirmed the clinical results of previous studies on IB-UCB transplant [9, 20], in terms of engraftment, platelet recovery and low incidence of GVHD. The cohort of the study was represented by very advanced patients, as displayed by the relatively high number of second transplants and refractory diseases. The negative patient selection importantly reduced the capacity of the study to unravel the anti-leukemic effect of UCB transplant, which is clearly stronger in MRD positive patients than in those with high burden of disease at transplant. Notably all the patients who had been transplanted in CR maintained the remission status after transplant and three of those transplanted with active disease were still in complete remission at last follow up. Although the highly pre-transplant treatments, NRM in the study was relatively low, with reduced risk of graft failure and GVHD. In this study we performed IB-UCB transplant using low dose ATLG and we found a satisfactory immune reconstitution, leading to acceptable infective morbidity and mortality. In particular, CD3 + T cells recovery was faster than previously described after intra-venous standard UCB transplant [21–24], due to better CD8 + T cells recovery. Although ATLG increases the risk of CMV infection, the rate of CMV reactivation in this study was similar to other reports [21, 25–27], but none of the patients either developed a CMV disease or died for CMV infection.

Owing to the study design, we are not able to ascertain whether the low incidence of GVHD depends upon the use of polyclonal serum or upon the route of transplant. The use of ATLG in the UCB transplant is still a matter of debate [21, 22, 24, 28]. Recently a pharmaco-kinetic/dynamic approach to ATLG dosing has been proposed, underpinning the importance of target cells burden and ATLG infusion [21–23, 29] timing. The ATLG brand and schedule administered to the IB-UCB patients in this trial is different from other published trials [21–24, 28, 29] due to its early timing (from −6 to −2 days) and dose (15–30 mg/Kg), which is expected to exert a strong anti-rejection rather than anti-GVHD activity. Nevertheless, the present ATLG schedule is still active in reducing GVHD [30, 31], without increasing infectious morbidity and mortality. However, our study confirms a dose-dependent effect of ATLG on T-cells recovery after UCB transplant as previously reported [21] (see Fig. 2).

Despite the good clinical performance of IB-UCB, new biological insight regarding the benefit of directly delivering HSC into the bone marrow niche has been provided. In this regard, a significant association between the hematologic (platelets) recovery and c-Mpl mRNA level, measured in CD34 + cells recovered on day + 10 after transplant, was found. Such c-Mpl expression level in CD34 + cells at day + 10 also positively correlates with the amount of CD34 + cells on day + 30 after IB-UCB transplant. The c-Mpl pathway is a pivotal regulator of HSC self-renewal as well as thrombopoiesis, and is currently regarded as a major target to design hematopoietic-reconstituting drugs [10, 32]. Interestingly, to perform the above observation, we processed CD34 + under controlled hypoxia, which resembles that of BM aspirates (see supplementary figure 1A and ref. [16]). Regarding the in vivo oxygen tension it is worth noting that pO_2_ in the venous blood is about 11% which is higher to that found in the BM aspirates (about 7%) [16]. However, the pO_2_ in the BM reaches extremely low levels, up to less that 1% in the endosteal niche [32, 33]. Accordingly, in vitro hypoxia improves the marrow repopulation efficiency of human UCB-HSC [15]. In line with these data, exposure to high oxygen content under hyperbaric conditions reduces the UCB-HSC proliferation, differentiation capability [34]. Hence, hypoxia is one of the most important micro-environmental factors that maintains and promotes HSC functional status [13, 17, 33–35]. These data suggest that out of the hypoxic BM niche, HSC lose part of their c-Mpl-driven HSC potential, due to exposure to atmospheric oxygen tension: the phenomenon may occur during ex vivo processing, as well as after intra-venous administration. On the contrary, chronic inflammation, a widely acknowledged age related condition [36], has been recently reported to negatively impinge upon c-Mpl signaling pathway activation [37]. Our data suggest that one of the benefits of the IB route of CD34 + cells injection may be the preservation of c-Mpl expression. Owing to the pivotal role of c-Mpl in thrombopoiesis, the above observations may also contribute to explain the remarkable recovery of platelets that occur in the IB-UCB transplant. Noteworthy, the quality and the speed of platelet engraftment have been already demonstrated to be predictive markers of good outcome after allotransplant [38]. Anyway, the fast platelet recovery after IB-UCB transplant without any ex vivo manipulation was very similar to the original report from Frassoni et al. [9].

The choice to perform UCB infusion in operating room, albeit it added up additional costs for the transplant procedure, was based on safety reasons and on the report by Frassoni et al. [9], which was the only study published at the time of protocol approval. However, the feasibility of IB infusion at bedside was subsequently reported by Murata et al. [39], thus improving the cost effectiveness of the IB procedure.

In this study, the amount of infused CD34 + cells correlates with hematological recovery and survival. Notably, since thawed CD34 + are highly correlated with CD34 + cells at cryopreservation, this latter maintains its role in the choice of the best UCB unit.

The major limitations of this study are the small size and the poor prognosis of the enrolled patients: both factors can impair the anti-leukemic effect of UCB.

Since that the number of infused CD34 + cells is substantially lower than that reported in the literature [40], it can be speculated that the high engraftment performance here reported may depend upon the IB route of infusion. However, because the number of infused TNC is similar to the standard UCB transplant, we cannot exclude that our results could be achieved by i.v. infusion.

However, the acceptable NRM and the fast platelet recovery justify a further investigation of IB transplant recruiting patients with more favorable prognosis. The correlation between the hematological recovery and c-Mpl expression, underlying the biological link with the hypoxic driven pathways occurring in the microenvironment after transplant, need also to be expanded to understand the molecular pattern linking c-Mpl pathway, hypoxia and hematopoietic recovery [41].

## Electronic supplementary material

Supplementary table I

Supplementary table II

Supplementary table III

Supplementary figure 1

Supplementary figure 2
