# Supplementary material for: Intrabone transplant provides full stemness of cord blood stem cells with fast hematopoietic recovery and low GVHD rate: results from a prospective study
Source: Bone Marrow Transplant. 2018 Sep 19;54(5):717–25. doi: 10.1038/s41409-018-0335-x (PMC6760547; doi:10.1038/s41409-018-0335-x)
Supplement: Supplementary file 1 — Supplementary table I [file 41409_2018_335_MOESM1_ESM.docx]

|  | **Supplementary table I Cell doses, HLA and blood mismatching of transplanted UCB units** | | | | | | | | | | |  |  |  |  |  |
| --- | --- | --- | --- | --- | --- | --- | --- | --- | --- | --- | --- | --- | --- | --- | --- | --- |
| **UPN** | **patient’s age** | **patient’s weight** | **pre** | **post thawing** | **pre** | **post** | **viability** | **volume of CB IB injected** | **HLA mismatches*** | | **blood compatibility** |  |  |  |  |  |
|  |  | **kg** | **thawing TNCx10^7^/kg** | **TNCx10^7^/kg** | **thawing** | **thawing** | **%** | **ml** |  |  |  |  |  |  |  |  |
|  |  |  |  |  | **CD34+x10^5^/kg** | **CD34+x10^5^/kg** |  |  | **class I** | **class II** |  |  |  |  |  |  |
| 762 | 29 | 52 | 3,1 | 1,82 | 1,29 | 0,4 | 57 | 28,5 | 1 | 1 | comp. |  |  |  |  |  |
| 767 | 54 | 80 | 1,91 | 1,76 | 0,12 | 0,23 | 95 | 19,5 | 2 | / | major inc. |  |  |  |  |  |
| 736 | 32 | 65 | 2,37 | 2,06 | 2,58 | 2,36 | 94 | 48 | 1 | 1 | comp. |  |  |  |  |  |
| 808 | 33 | 59,5 | 2,91 | 2,37 | 1,43 | 1,02 | 96 | 27,4 | 2 | / | minor inc. |  |  |  |  |  |
| 812 | 27 | 53 | 3,64 | 3,1 | 0,68 | 0,47 | 92 | 28,8 | 1 | / | major inc. |  |  |  |  |  |
| 816 | 41 | 54 | 3,04 | 1,02 | 0,76 | 0,23 | 37 | 19 | 2 | / | comp. |  |  |  |  |  |
| 752 | 36 | 60 | 4,75 | 3,92 | 2,26 | 1,61 | 95 | 28 | 2 | / | minor inc. |  |  |  |  |  |
| 822 | 34 | 93 | 1,94 | 1,4 | 0,82 | 0,46 | 75 | 23,1 | 2 | / | comp. |  |  |  |  |  |
| 832 | 50 | 56 | 4,13 | 3,7 | 3,46 | 2,9 | 81 | 23,8 | 2 | / | minor inc. |  |  |  |  |  |
| 835 | 43 | 54 | 5,48 | 2,9 | 1,84 | 0,64 | 91 | 28,7 | 2 | / | comp. |  |  |  |  |  |
| 848 | 35 | 63 | 3,02 | 2,02 | 2,26 | 1,5 | 72 | 29 | 1 | / | minor inc. |  |  |  |  |  |
| 849 | 34 | 70 | 2,72 | 1,93 | 0,71 | 0,41 | 96 | 50,2 | 1 | 1 | minor inc. |  |  |  |  |  |
| 855 | 25 | 54 | 4,06 | 2,9 | 1,93 | 1,67 | 89 | 23,4 | 1 | 1 | minor inc. |  |  |  |  |  |
| 862 | 18 | 67 | 2,97 | 2,44 | 0,76 | 0,37 | 95 | 31,4 | 1 | 1 | major inc. |  |  |  |  |  |
| 892 | 50 | 78 | 2,33 | 1,95 | 1,07 | 0,35 | 96 | 43,5 | 1 | / | major inc. |  |  |  |  |  |
| 864 | 50 | 66 | 2,86 | 2,18 | 0,75 | 0,41 | 96 | 23,6 | 2 | / | major inc. |  |  |  |  |  |
| 897 | 43 | 59,5 | 3,34 | 1,95 | 0,55 | 0,23 | 99 | 27,6 | 2 | / | minor inc. |  |  |  |  |  |
| 727 | 54 | 55 | 3,93 | 3,8 | 1,4 | 1,44 | 95 | 26 | 2 | / | comp. |  |  |  |  |  |
| 903 | 36 | 87,5 | 2,93 | 1,79 | 1,06 | 0,538 | 65 | 29,1 | 2 | / | major inc. |  |  |  |  |  |
| 906 | 48 | 65 | 3,41 | 2,26 | 1,87 | 1,265 | 52 | 27 | 2 | / | major inc. |  |  |  |  |  |
| 908 | 43 | 69 | 4,54 | 3,3 | 1,79 | 0,74 | 70 | 26,2 | 1 | 1 | major inc. |  |  |  |  |  |
| 910 | 27 | 72,8 | 3,57 | 0,2 | 3,27 | 1,72 | 73 | 58,2 | 1 | 1 | major inc. |  |  |  |  |  |
| 909 | 56 | 58,5 | 2,73 | 1,74 | 0,83 | 0,347 | 67 | 25,6 | 2 | / | major inc. |  |  |  |  |  |
|  |  |  |  |  |  |  |  |  |  |  |  |  |  |  |  |  |
| Median (range) | 36 | 63 | 3,04 | 2,06 |  | 0,54 | 91 | 27,6 |  |  |  |  |  |  |  |  |
|  | (18-56) | (52-93) | (1,91-5,48) | (0,20-3,92) | 1,29 | (0,23-2,90) | (37-99) | (19,0-58,2) |  |  |  |  |  |  |  |  |
|  |  |  |  |  | (0,12-3,46) |  |  |  |  |  |  |  |  |  |  |  |
|  |  |  |  |  |  |  |  |  |  |  |  |  |  |  |  |  |
| Footnote: TNC: total nucleated cells; IB: intrabone; comp. : ABO compatibility; minor inc.: minor ABO incompatibility; major inc.: major ABO incompatibility. | | | | | | | | | | | | | |  |  |  |
| *HLA mismatches: class I mismatches are antigenic while those in DRB1 are always at allelic level, according to the Italian Bone Marrow Donor Registry’s  (IBMDR) standard criteria. | | | | | | | | | | | | | | | | |
