# Supplementary material for: Intrabone transplant provides full stemness of cord blood stem cells with fast hematopoietic recovery and low GVHD rate: results from a prospective study
Source: Bone Marrow Transplant. 2018 Sep 19;54(5):717–25. doi: 10.1038/s41409-018-0335-x (PMC6760547; doi:10.1038/s41409-018-0335-x)
Supplement: Supplementary file 2 — Supplementary table II [file 41409_2018_335_MOESM2_ESM.docx]

| **Supplementary table II. Univariate analysis for overall survival (OS), relapse incidence (RI), non-relapse mortality (NRM), GVHD and hematologic recovery** | | | | | | |
| --- | --- | --- | --- | --- | --- | --- |
|  | **2y OS** | **2y CI of relapse** | **2y CI of NRM** | **aGVHD>II at D100** | **ANC>0.5x 10^9^/L at day 60** | **Platelet> 0.5x 10^9^/L at day 90** |
| Status at Tx |  |  |  |  |  |  |
| no CR1-2 | 17%+14% | 53%+13% | 20%+11% | 25%+17% | 78%+12% | 79%+12% |
| CR1-2 | 58%+25% | 14%+14% | 13%+12% | 7%+6% | 88%+17% | 88%+18% |
| p-value | 0.40 | 0.09 | 0.98 | 0.2 | 0.42 | 0.26 |
|  |  |  |  |  |  |  |
| TNC infused |  |  |  |  |  |  |
| <median | 24%+19% | 39%+17% | 28%+15% | 17%+11% | 83%+14% | 83%+14% |
| >median | 33%+18% | 68%+23% | 13%+13% | 9%+9% | 80%+15% | 80%+18% |
| p-value | 0.6 | 0.46 | 0.28 | 0.64 | 0.69 | 0.73 |
|  |  |  |  |  |  |  |
| CD34+ cells infused |  |  |  |  |  |  |
| <median | 38%+18% | 36%+16% | 36%+15% | 17%+11% | 75%+14% | 75%+13% |
| >median | 30%+18% | 77%+25% | 0% | 9%+9% | 90%+12% | 90%+12% |
| p-value | 0.74 | 0.27 | 0.055 | 0.64 | **0.04** | **0.007** |
|  |  |  |  |  |  |  |
| CD3+ cells infused |  |  |  |  |  |  |
| <median | 26%+20% | 46%+20% | 24%+17% | 18%+12% | 91%+13% | 91%+9% |
| >median | 40%+18% | 69%+24% | 10%+10% | 9%+9% | 80%+15% | 80%+15% |
| p-value | 0.47 | 0.71 | 0.35 | 0.59 | 0.54 | 0.34 |
|  |  |  |  |  |  |  |
| TNC at cryopr. |  |  |  |  |  |  |
| <median | 65%+14% | 58%+19% | 27%+15% | 20%+14% | 83%+14% | 75%+14% |
| >median | 65%+14% | 45%+20% | 9%+8% | 9%+9% | 80%+15% | 64%+16% |
| p-value | 0.52 | 0.79 | 0.41 | 0.74 | 0.69 | 0.80 |
|  |  |  |  |  |  |  |
| CD34+ at cryopr. |  |  |  |  |  |  |
| <median | 44%+20% | 36%+16% | 36%+15% | 21%+14% | 75%+14% | 58%+16% |
| >median | 25%+16% | 77%+25% | 0 | 9%+9% | 90%+12% | 82%+13% |
| p-value | 0.65 | 0.27 | 0.055 | 0.68 | **0.008** | **0.09** |
|  |  |  |  |  |  |  |
| Age |  |  |  |  |  |  |
| <median | 28%+14% | 63%+17% | 27%+15% | 17%+11% | 72%+15% | 73%+15% |
| >median | 39%+28% | 31%+16% | 9%+9% | 9%+9% | 91%+13% | 91%+9% |
| p-value | 0.32 | 0.28 | 0.43 | 0.63 | 0.6 | 0.88 |
|  |  |  |  |  |  |  |
| ATG |  |  |  |  |  |  |
| 15 mg/kg | 83%+15% | 11%+11% | 18%+17% | 22%+15% | 100% | 100% |
| 30 mg/kg | 27%+12% | 64%+14% | 21%+12% | 7%+7% | 69%+14% | 69%+14% |
| p-value | 0.25 | 0.09 | 0.71 | 0.31 | 0.25 | 0.49 |
|  |  |  |  |  |  |  |
| Footnote: CI: cumulative incidence; CR1-2: first or second complete remission at transplant; TNC: total nucleated cells; D60: day 60 after transplant;  D90: day 90 after transplant; cryopr.: cryopreservation. Significant p values are in bold. | | | | | | |
