# Supplementary material for: Intrabone transplant provides full stemness of cord blood stem cells with fast hematopoietic recovery and low GVHD rate: results from a prospective study
Source: Bone Marrow Transplant. 2018 Sep 19;54(5):717–25. doi: 10.1038/s41409-018-0335-x (PMC6760547; doi:10.1038/s41409-018-0335-x)
Supplement: Supplementary file 3 — Supplementary table III [file 41409_2018_335_MOESM3_ESM.docx]

| **Supplementary table III. Multivariate analysis for hematologic recovery, overall survival (OS), relapse incidence (RI) and non relapse mortality (NRM)** | | | | | | | | | | | | | | | | | |  | |  |
| --- | --- | --- | --- | --- | --- | --- | --- | --- | --- | --- | --- | --- | --- | --- | --- | --- | --- | --- | --- | --- |
|  |  | | | | ***p value*** | ***HR*** | ***95% Confidence Interval*** | | | | |  | |  | |  | |  | |  |
|  |  |  |  |  |  |  |  | | | | |  | |  | |  | |  | |  |
|  |  | | | |  |  |  | |  | | |  | |  | |  | |  | |  |
| ANC>0.5x 10^9^/L | CD34+ cells>median vs < median | | | | **0.006** | 5.04 | 1.59 | | 15.94 | | |  | |  | |  | |  | |  |
|  | CR1-CR2 vs other | | | | 0.89 | 1.07 | 0.44 | | 2.58 | | |  | |  | |  | |  | |  |
|  | age>median vs < median | | | | 0.37 | 1.64 | 0.56 | | 4.86 | | |  | |  | |  | |  | |  |
|  | ATG 30 mg vs 15 mg | | | | 0.26 | 0.97 | 0.91 | | 1.03 | | |  | |  | |  | |  | |  |
|  |  | | | |  |  |  | |  | | |  | |  | |  | |  | |  |
| Platelet>0.5x 10^9^/L | CD34+ cells>median vs < median | | | | **0.02** | 4.58 | 1.27 | | 16.62 | | |  | |  | |  | |  | |  |
|  | CR1-CR2 vs other | | | | 0.25 | 1.64 | 0.71 | | 3.79 | | |  | |  | |  | |  | |  |
|  | age>median | | | | 0.33 | 2.26 | 0.44 | | 11.7 | | |  | |  | |  | |  | |  |
|  | ATG 30 mg vs 15 mg | | | | 0.32 | 0.96 | 0.87 | | 1.05 | | |  | |  | |  | |  | |  |
|  |  | | | |  |  |  | |  | | |  | |  | |  | |  | |  |
| Overall survival (OS) | CD34>median vs < median | | | | 0.62 | 0.73 | 0.21 | | 2.53 | | |  | |  | |  | |  | |  |
|  | CR1-CR2 vs other | | | | 0.44 | 0.54 | 0.12 | | 2.56 | | |  | |  | |  | |  | |  |
|  | age>median | | | | 0.68 | 0.74 | 0.17 | | 3.2 | | |  | |  | |  | |  | |  |
|  | ATG 30 mg vs 15 mg | | | | 0.37 | 1.07 | 0.92 | | 1.26 | | |  | |  | |  | |  | |  |
|  |  | | | |  |  |  | |  | | |  | |  | |  | |  | |  |
| Relapse incidence (RI) | CD34>median vs < median | | | | 0.19 | 2.08 | 0.7 | | 6.15 | | |  | |  | |  | |  | |  |
|  | CR1-CR2 vs other | | | | 0.15 | 0.17 | 0.02 | | 1.86 | | |  | |  | |  | |  | |  |
|  | age>median | | | | 0.78 | 1.2 | 0.33 | | 4.33 | | |  | |  | |  | |  | |  |
|  | ATG 30 mg vs 15 mg | | | | 0.11 | 1.12 | 0.97 | | 1.28 | | |  | |  | |  | |  | |  |
|  |  | | | |  |  |  | |  | | |  | |  | |  | |  | |  |
| Non-relapse mortality (NRM) | | | NA (no event in 1 group) | |  |  |  | |  | | |  | |  | |  | |  | |  |
|  |  |  | |  |  |  | |  | |  |  | |  | |  | |  | |  | |
| Footnote: CR1-2: first or second complete remission at transplant; TNC: total nucleated cells; ATG: antilymphocyte globulin. Significant p values are in bold. | | | | | | | | | | | | | | | |  | |  | |  |
