# Supplementary material for: Intrabone transplant provides full stemness of cord blood stem cells with fast hematopoietic recovery and low GVHD rate: results from a prospective study
Source: Bone Marrow Transplant. 2018 Sep 19;54(5):717–25. doi: 10.1038/s41409-018-0335-x (PMC6760547; doi:10.1038/s41409-018-0335-x)
Supplement: Supplementary file 4 — Supplementary figure 1 [file 41409_2018_335_MOESM4_ESM.pdf]

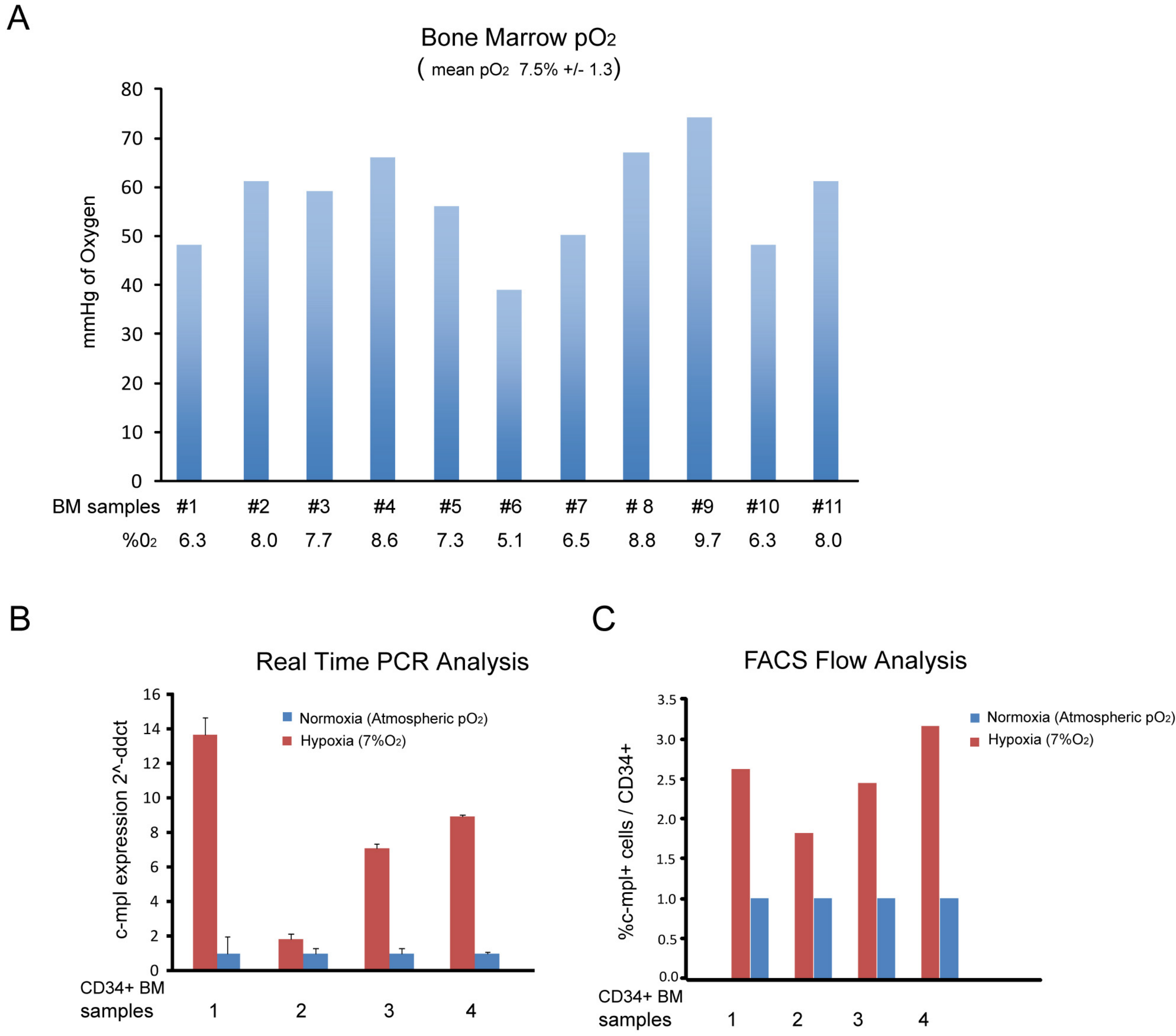

Figure legend:

A, pO_2 level in 11 BM aspirates by means of blood gas analysis syringe;

B, Real time PCR analysis of c-Mpl expression in CD34+ cells isolated in hypoxic (7% pO_2) or normoxic (20% pO_2) conditions;

C, Percentage of c-Mpl positive cells on CD34+ cells isolated in hypoxic (7% pO_2) or normoxic (20% pO_2) conditions;

Bonifazi et al_Supplementary Figure 1
