# Supplementary material for: Intrabone transplant provides full stemness of cord blood stem cells with fast hematopoietic recovery and low GVHD rate: results from a prospective study
Source: Bone Marrow Transplant. 2018 Sep 19;54(5):717–25. doi: 10.1038/s41409-018-0335-x (PMC6760547; doi:10.1038/s41409-018-0335-x)
Supplement: Supplementary file 5 — Supplementary figure 2 [file 41409_2018_335_MOESM5_ESM.pdf]

A

CXCR4

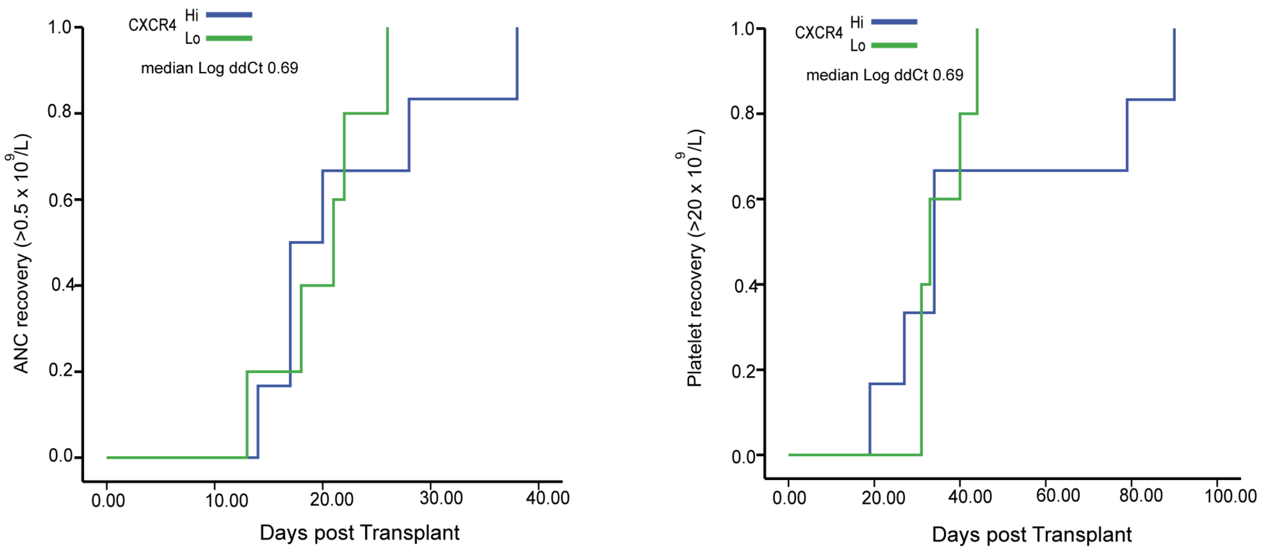

B

NOTCH1

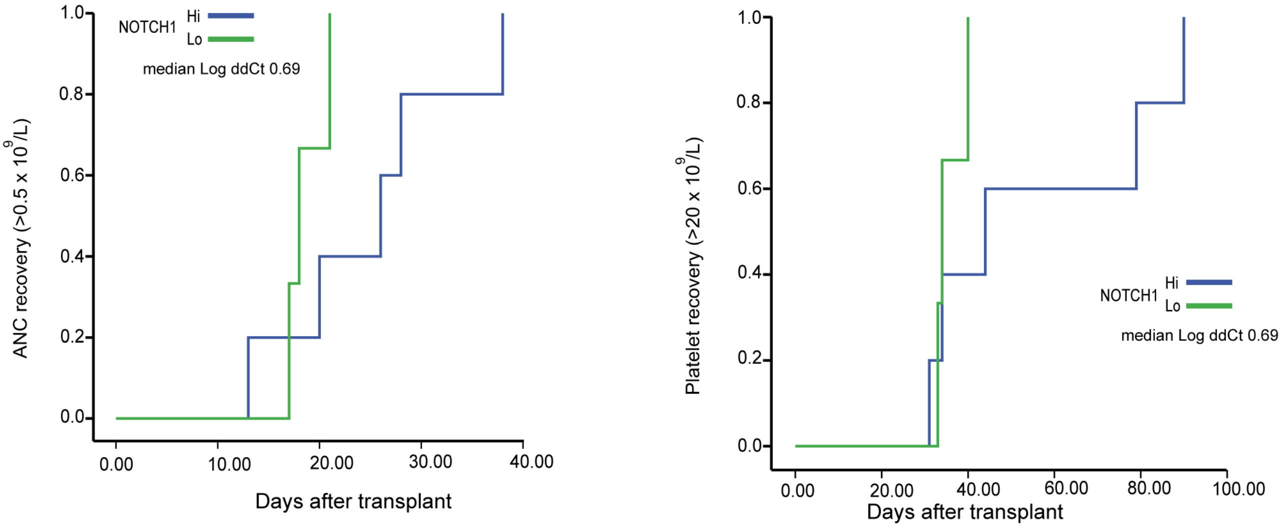

Figure legend

A, Hematological recovery (ANC $>0.5x10^9$/L, left panel, platelet $>20x10^9$/L, right panel) according to the expression of CXCR4 at day +10 in the CD34+ cells isolated under hypoxic conditions. No statistical association was found;

B, Hematological recovery (ANC $>0.5x10^9$/L, left panel, platelet $>20x10^9$/L, right panel) according to the expression of Notch-1 at day +10 in the CD34+ cells isolated under hypoxic conditions. No statistical association was found;

Bonifazi et al., Supplementary Fig 2
